# Abnormalities in ileal stem, neurogenin 3, and enteroendocrine cells in patients with irritable bowel syndrome

**DOI:** 10.1186/s12876-017-0643-4

**Published:** 2017-08-01

**Authors:** Magdy El-Salhy, Odd Helge Gilja

**Affiliations:** 1 Section for Gastroenterology, Department of Medicine, Stord Helse-Fonna Hospital, Box 4000, 54 09 Stord, Stord, Norway; 2 Section for Gastroenterology, Department of Clinical Medicine, University of Bergen, Bergen, Norway; 3 National Centre for Functional Gastrointestinal Disorders, Department of Medicine, Haukeland University Hospital, Bergen, Norway; 4 National Centre for Ultrasound in Gastroenterology, Department of Medicine, Haukeland University Hospital, Bergen, Norway

**Keywords:** Enteroendocrine cells, IBS, Ileum, Immunohistochemistry, Mushasi-1, Neurogenin 3, PYY, Serotonin

## Abstract

**Background:**

This study examined whether the densities of stem- and enteroendocrine cell progenitors are abnormal in the ileum of patients with irritable bowel syndrome (IBS), and whether any abnormalities in ileal enteroendocrine cells are correlated with abnormalities in stem cells and enteroendocrine cell progenitors.

**Methods:**

One hundred and one IBS patients covering all IBS subtypes were recruited, and 39 non-IBS subjects were included as a control group. The patients and controls underwent standard colonoscopies, during which biopsy specimens were obtained from the ileum. The biopsy specimens were stained with hematoxylin-eosin and immunostained for Musashi-1 (Msi-1), neurogenin 3 (NEUROG3), chromogranin A (CgA), serotonin, peptide YY (PYY), oxyntomodulin (enteroglucagon), pancreatic polypeptide, and somatostatin. The immunoreactive cells were quantified by computerized image analysis.

**Results:**

The densities of Msi-1, NEUROG3, CgA, and serotonin cells were reduced in all IBS patients and in patients with diarrhea-predominant IBS (IBS-D), mixed-diarrhea-and-constipation IBS (IBS-M), and constipation-predominant (IBS-C) relative to the control subjects. While the PYY cell density was increased in IBS-C relative to controls, it did not differ between control subjects and IBS-D and IBS-M patients. The densities of Msi-1 and NEUROG3 cells were strongly correlated with that of CgA cells.

**Conclusions:**

The abnormalities in the ileal enteroendocrine cells appear to be caused by two mechanisms: (1) decreases in the clonogenic activity of the stem cells and in the endocrine-cell progenitors differentiating into enteroendocrine cells, and (2) switching on the expression of PYY and switching off the expression of certain other hormones in other types of the enteroendocrine cells.

## Background

Irritable bowel syndrome (IBS) is a chronic gastrointestinal condition with a prevalence of 5–20% of the world adult population [1–9]. IBS is reportedly also present in in about 50% of patients with ulcerative colitis patients and Crohn’s disease in remission [10, 11]. Furthermore, 38% of patients with celiac disease suffer from IBS symptoms despite consuming a gluten-free diet [12].

The etiology of IBS is unclear, but certain factors appear to play a pivotal role in its pathophysiology, including genetic factors, intestinal bacterial flora, diet, and chronic low-grade intestinal inflammation [13, 14]. Abnormally low densities of endocrine cells in the stomach, duodenum, ileum, colon, and rectum have been reported in patients with IBS [15–29], which could explain the dysmotility, visceral hypersensitivity, and abnormal secretion seen in IBS patients [30]. These observations have prompted suggestions that the abnormalities in the gut endocrine cells play a significant role in the pathophysiology of IBS [13, 14].

Musashi-1 (Msi-1) is localized in gastrointestinal stem cells and their early progenitors, and neurogenin 3 (NEUROG3) is a marker for early intestinal endocrine-cell progenitors [31–33]. It has been reported recently that the densities of Msi-1 and NEUROG3 cells are reduced in the duodenum of patients with IBS, with these decreases being associated with reductions in duodenal endocrine cells, which could in turn be caused by decreases in stem cells and their proliferation progeny into endocrine cells [34].

The types and densities of endocrine cells differ between the distal and proximal parts of the small intestine, which is probably due to the quite different functions of these two sections of the gastrointestinal tract [35, 36]. Whereas the proximal small intestine contains serotonin, secretin, cholecystokinin, gastric inhibitory polypeptide (GIP), and somatostatin cells, the distal small intestine contains serotonin, peptide YY (PYY), pancreatic polypeptide (PP), oxyntomodulin (enteroglucagon), and somatostatin cells [35, 36].

This study aimed at examining whether the densities of stem- and endocrine-cell progenitors as manifested by Msi-1 and NEUROG3 are affected in the ileum of patients with IBS. Furthermore, the densities of ileal endocrine cells were measured with the aim of establishing their correlation with possible abnormalities in stem- and endocrine-cell progenitors.

## Methods

### Patients and controls

One hundred and one patients with IBS according to Rome III criteria [37, 38] were recruited at Stord Hospital, Stord, Norway. These patients comprised 80 females and 21 males with a mean age of 40 years (age range 18–65 years). Thirty-five of the patients had diarrhea-predominant IBS (IBS-D), 34 had mixed-diarrhea-and-constipation IBS (IBS-M), and 32 had constipation-predominant IBS (IBS-C). The IBS symptoms had been present in all of the patients for many years, and their onset was not identified as being associated with gastrointestinal infection. All of the patients underwent a physical examination and were investigated by blood tests to exclude inflammatory, endocrine, liver, kidney, and systemic diseases. Furthermore, celiac disease was excluded by performing histopathological examinations of duodenal biopsy samples obtained during gastroscopy.

A control group was included that comprised 39 non-IBS subjects (27 females and 12 males; mean age 38 years, age range 18–64 years) who had undergone colonoscopies because of (1) gastrointestinal bleeding that was found to be due to hemorrhoids (*n* = 24) or angiodysplasia (*n* = 3), or (2) health worries resulting from a relative being diagnosed with colon carcinoma (*n* = 12).

The study was performed in accordance with the Declaration of Helsinki and was approved by the Regional Committee for Medical and Health Research Ethics West, Bergen, Norway. All subjects gave both oral and written consents to participate.

### Colonoscopy, histopathology, and immunohistochemistry

A standard colonoscopy was performed on both the patients and controls, with segmental biopsy specimens taken from the colon and rectum, and four biopsy samples taken from the ileum of each subject. The biopsy samples were fixed overnight in 4% buffered paraformaldehyde, embedded in paraffin, and cut into 5-μm-thick sections. The sections were stained with hematoxylin-eosin, and immunostained using the ultraView Universal DAB Detection Kit (version 1.02.0018, Venata Medical Systems, Basel, Switzerland) and the BenchMark Ultra IHC/ISH staining module (Venata Medical Systems). The sections were incubated with the primary antibodies for 30 min at 37 °C. Details of the primary antibodies used are given in Table 1.

**Table 1:** Primary antibodies used for immunohistochemical staining

Antibody target	Source	Code number	Working dilution	Type of antibody
Msi-1	Novus Biologicals Europe (Abingdon, UK)	NB100–1759	1:100	Polyclonal, raised in rabbit against residues 5–21 [APQPGLASPDSPHDPCK] of human, mouse, and rat Msi-1
NEUROG3	ThermoFisher Scientific (Oslo, Norway)	BT-B56180	1:50	Polyclonal, raised in rabbit against KLH-conjugated synthetic peptide at 40–69 amino acids from the N-terminal region of human NEUROG3
CgA	Dako (Glostrup, Denmark)	M869	1:1000	Monoclonal, raised in mouse against the N-terminal of purified CgA
Serotonin	Dako	5HT-209	1:1500	Monoclonal, raised in mouse against serotonin
PYY	Alpha-Diagnostica (San Antonio, TX, USA)	PYY 11A	1:1000	Polyclonal, raised in rabbit against PYY
Oxyntomodulin (enteroglucagon)	Acris Antibodies (Herford, Germany)	BP508	1:800	Polyclonal, raised in rabbit against porcine glicentin/glucagon
PP	Diagnostic Biosystems (Pleasanton, CA, USA)	#114	1:400	Polyclonal, raised in rabbit against synthetic human PP
Somatostatin	Dako	A566	1:200	Polyclonal, raised in rabbit against synthetic human somatostatin

### Computerized image analysis

Morphometric measurements were performed by applying imaging software (version 1.7, cellSens, Olympus) to images obtained with the aid of a computer linked to a microscope (type BX 43, Olympus) with a camera (DP 26, Olympus). The number of immunoreactive cells and the area of the epithelial cells were measured. The numbers of Msi-1, NEUROG3, and endocrine cells in each field were counted manually by pointing and clicking the computer mouse. The area of epithelial cells was determined by manually drawing an enclosed region using the computer mouse. A × 40 objective was used, which resulted in each frame (field) on the monitor representing a tissue area of 0.14 mm^2^. The density of endocrine cells was expressed as the number of endocrine cells per square millimeter of epithelium, the density of Msi-1 cells was expressed as the number of immunoreactive cells per crypt, and the density of NEUROG3 cells was expressed as the number of immunoreactive cells per field. Quantification was performed in ten randomly chosen microscopic fields. The measurements were made by the same person (M.E-S.) who was blind to the identities of the slides.

### Statistical analysis

Differences in the sex and age distributions between patients and controls were tested using Fisher’s exact test and the Mann-Whitney nonparametric test, respectively. Differences between control subjects, all IBS patients (IBS-total), and IBS-D, IBS-M, and IBS-C patients were tested using the Kruskal-Wallis nonparametric test with Dunn’s posttest. The Kruskal-Wallis test is a nonparametric test that compares three or more unmatched groups, while Dunn’s multiple-comparisons test compares the difference in the sum of ranks between two columns with the expected average difference. Correlations were assessed using the Spearman nonparametric test. The data are presented as mean ± SEM values, and differences for which *P* < 0.05 were considered to be statistically significant.

## Results

### Sex and age characteristics of patients and controls

The sex and age distributions did not differ significantly between the patients and controls (*P* = 0.27 and *P* = 0.67, respectively).

### Endoscopy, histopathology, and immunohistochemistry

The ileum, colon, and rectum of both the patients and control subjects were macroscopically normal. The findings of histopathological examinations of the ileum, colon, and rectum were also normal in both the patients and controls.

Cells that were immunoreactive for Msi-1, NEUROG3, chromogranin A (CgA), serotonin, PYY, PP, oxyntomodulin (enteroglucagon), and somatostatin were found in the ileum of all of the subjects (i.e., both patients and controls), mostly in the crypts. These cells were basket- or flask-shaped, and sometimes had a basal cytoplasmic process.

### Computerized image analysis

**Fig. 1:**
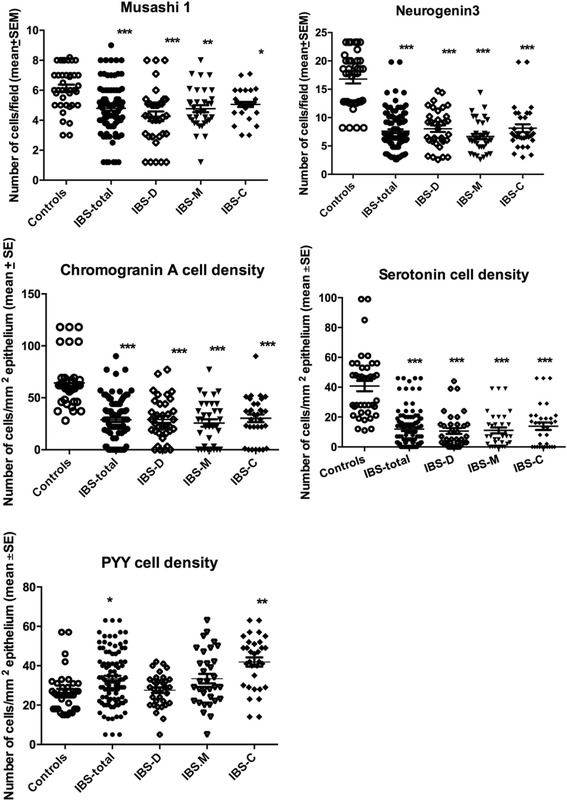
Densities of Msi-1, NEUROG3, CgA, serotonin, and PYY cells in the ileum of control subjects and IBS-total, IBS-D, IBS-M, and IBS-C patients. *P < 0.05, **P < 0.01, and ***P < 0.001 vs. controls (Dunn’s multiple-comparisons test)

**Fig. 2:**
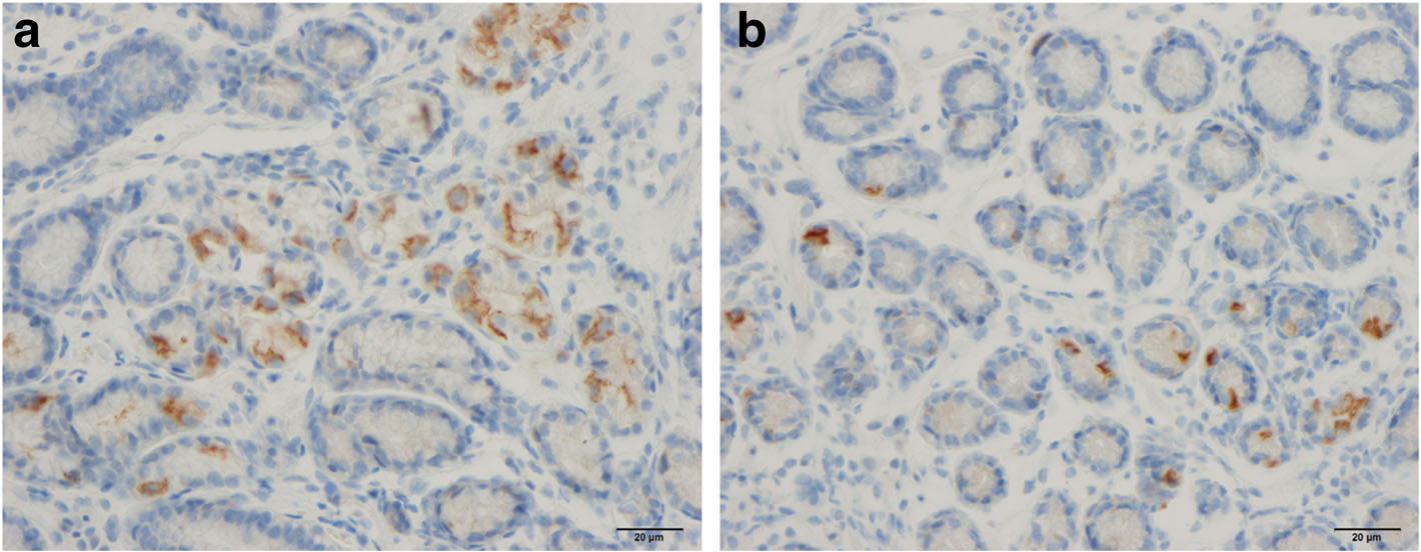
Ileal Msi-1 cells in a control subject (**a**) and an IBS patient (**b**)

**Fig. 3:**
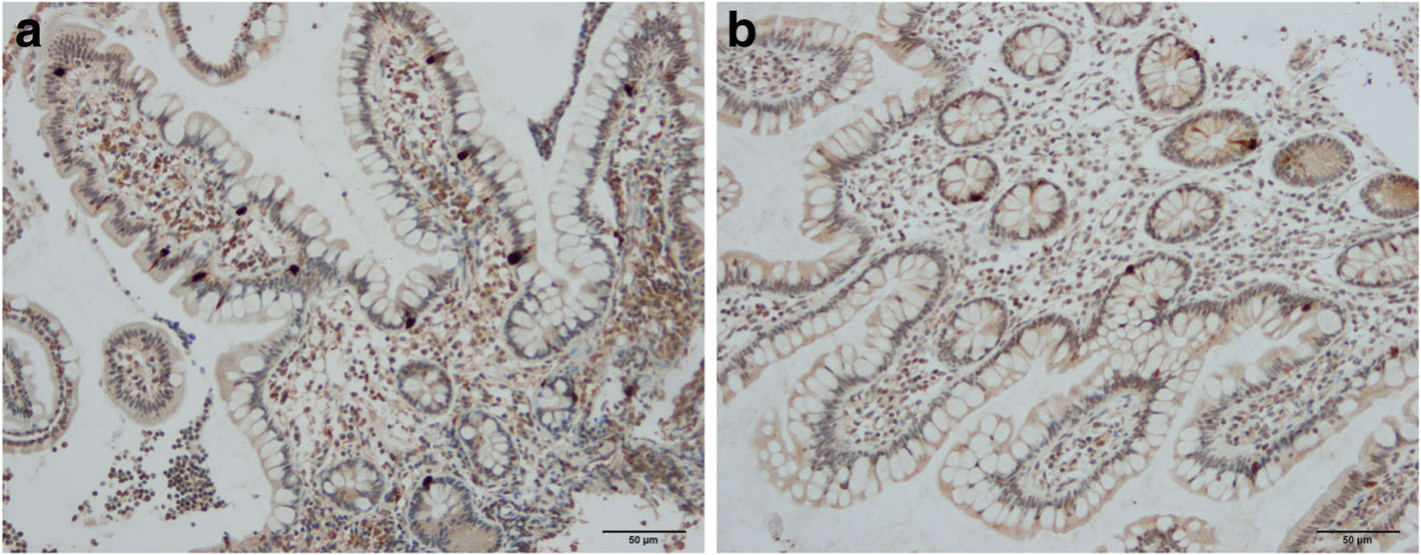
Ileal NEUROG3 cells in a control subject (**a**) and an IBS patient (**b**)

**Fig. 4:**
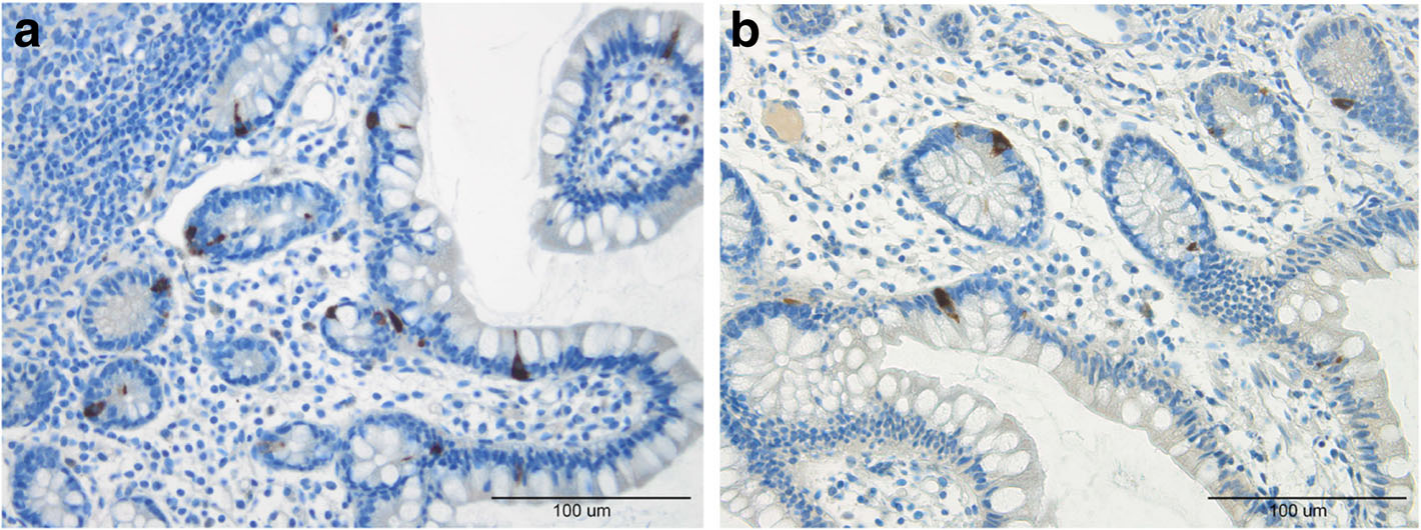
Ileal CgA cells in a control subject (**a**) and a patient with IBS (**b**)

**Fig. 5:**
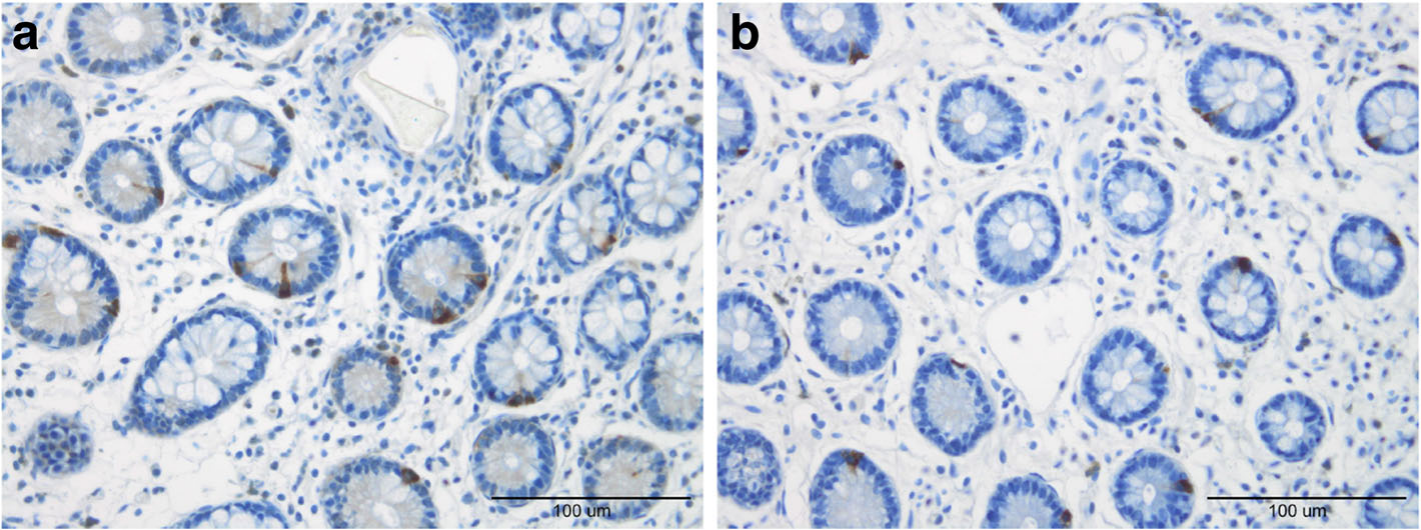
Ileal serotonin cells in a control subject (**a**) and a patient with IBS (**b**)

**Fig. 6:**
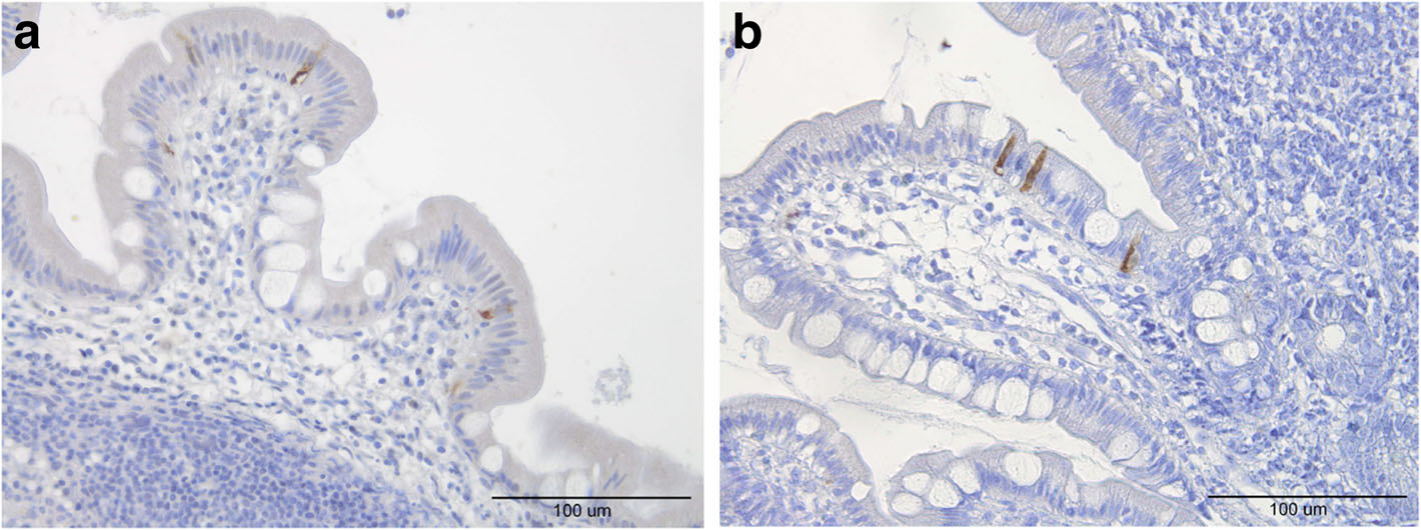
Ileal PYY cells in a control subject (**a**) and a patient with IBS-C (**b**)

The densities of Msi-1, NEUROG3, CgA, serotonin, and PYY cells are summarized in Table 2 and illustrated in Figs. 1, 2, 3, 4, 5 and 6. Few of the cells in the examined biopsy material were immunoreactive for PP, enteroglucagon, or somatostatin, which made it impossible to reliably quantify these types of cells.

**Table 2:** Densities of immunoreactive cells in control subjects, IBS-total patients, and IBSsubtype patients

Cell type	Controls	IBS-total	IBS-D	IBS-M	IBS-C
Msi-1	6.2 ± 0.2	4.8 ± 0.2***	4.3 ± 0.3***	4.8 ± 0.2**	5.1 ± 0.2*
NEUROG3	16.8 ± 0.8	7.6 ± 0.3***	8.0 ± 0.6***	6.7 ± 0.5***	8.1 ± 0.7***
CgA	64.3 ± 3.6	28.8 ± 2.11***	29.2 ± 3.5***	25.7 ± 3.6***	30.3 ± 3.7***
Serotonin	40.8 ± 3.5	12.0 ± 1.3***	10.7 ± 2.0***	11.1 ± 2.0***	13.9 ± 2.5***
PYY	26.6 ± 1.6	33.5 ± 1.4*	27.6 ± 1.4	33.5 ± 2.4	41.9 ± 2.3**

*P < 0.05, **P < 0.01, ***P < 0.001 vs. controls (Dunn’s multiple-comparisons test)

The Msi-1 cell density was significantly lower in IBS-total, IBS-D, IBS-M, and IBS-C patients than in the control subjects (*P* < 0.001, *P* < 0.001, *P* < 0.01, and *P* < 0.05, respectively). Dunn’s posttest showed that the NEUROG3 cell density was lower in IBS-total, IBS-D, IBS-M, and IBS-C patients than in the controls (*P* < 0.001 for all).

The Kruskal-Wallis nonparametric test showed significant differences in the CgA and serotonin cell densities between control subjects, IBS-total patients, and IBS-subtype patients (*P* < 0.0001 for both cell types). The CgA and serotonin cell densities were lower in IBS-total, IBS-D, IBS-M, and IBS-C patients than in controls (*P* < 0.001 for both cell types). The Kruskal-Wallis test revealed significant differences in PYY cell density between controls and IBS-total, IBS-D, IBS-M, and IBS-C patients (*P* < 0.0001 for all). Dunn’s posttest showed that the PYY cell density was significantly higher in IBS-total and IBS-C patients than in the control subjects (*P* < 0.02 and *P* < 0.01, respectively), while it did not differ significantly between IBS-D and IBS-M patients and the control subjects (*P* = 0.2 and *P* = 0.5, respectively).

**Fig. 7:**
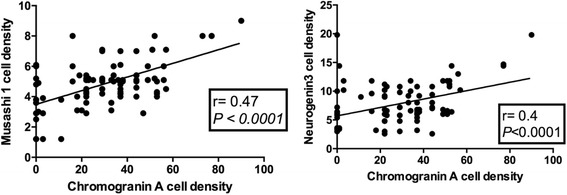
Correlations between the density of CgA cells and the densities of Msi-1 and NEUROG3 cells (Spearman nonparametric test)

The densities of Msi-1 and NEUROG3 cells were strongly correlated with that of CgA cells (*P* < 0.0001 and *r* = 0.47, and *P* < 0.0001 and *r* = 0.40, respectively) (Fig. 7).

## Discussion

Each crypt in the intestine contains four to six stem cells. Stem cells either divide into 2 new stem cells (clonogeny) or differentiate into mucosal epithelial cells (differentiation progeny) [33, 39–52]. The stem cell differentiation comprises two lineages: secretory and absorptive. The secretory lineage ends to goblet, endocrine, and Paneth cells, and the absorptive lineage leads to absorptive enterocytes [33, 39–52]. Msi-1 occurs in both intestinal stem cells and early progenitors [33, 53–55], while NEUROG3 is localized in intestinal endocrine-cell progenitors [32, 56, 57]. In the present study the densities of both Msi-1 and NEUROG3 cells in the ileum were lower in patients with IBS than in controls, which is similar to previous findings in the proximal small intestine [34].

CgA occurs in all types of enteroendocrine cells [58–60], with CgA cells density reflecting the total density of the ileal endocrine cells. Similar to the situation in the proximal small intestine [34], the CgA cells density in the ileum was reduced in the present IBS patients, which is consistent with previous observations that we have made in another cohort of patients [61]. The reduction in CgA cell density observed herein was significantly correlated with the reductions in Msi-1 and NEUROG3 cells. A reduction in NEUROG3 was associated with a decrease in the number of intestinal endocrine cells in patients with congenital malabsorptive diarrhea [31], in patients with small-intestine allograft rejection [32], and in NEUROG3-knockout mice [62]. It is tempting to speculate that the decreased density of ileal endocrine cells observed in the present IBS patients is caused by the reductions in Msi-1 and NEUROG3 cells.

Serotonin cells are the main endocrine cell type in the ileum [36]. The decreased cell density of CgA cell density found here and in our previous cohort of IBS patients [29] may be caused by the decreases in the serotonin cell density. Serotonin stimulates small-, and large intestine motility, and activates the submucosal enteric nervous system that transports sensation from the gastrointestinal tract to the central nervous system [30].

While the density of ileal PYY cells was similar to the control subjects in the IBS-D and IBS-M patients, it was higher in IBS-C patients, which is consistent with previously results obtained in another cohort of IBS patients [29]. It is well known that enteroendocrine cell can express two hormones, such as glucagon-like peptide-1 and GIP in the small intestine, and PYY and oxyntomodulin in the distal small and large intestines [63–65]. It has been shown further that mature enteroendocrine cells are able of synthetize up to seven different hormones [66–68]. The increase in the density of PYY cells in IBS-C is probably caused by switching off the expression of other hormones and switching on the expression of PYY. PYY regulates the so-called ileal brake and increases the absorption of water and electrolytes [69–74]. It also inhibits vasoactive intestinal polypeptide and prostaglandin E2, both of which stimulate intestinal fluid secretion [75–77]. It is possible that the increase in the PYY cell density in IBS-C patients would slow the intestinal transit by strengthening the ileal brake, increasing the absorption of water, and decreasing the secretion of the intestinal fluid, and thereby also result in constipation.

It is difficult to conclude whether the changes in stem and enteroendocrine cells observed in here are primary or secondary to IBS. However, it was hypothesized recently that factors known to play a part in the pathophysiology of IBS—including genetics, diet, the intestinal microbiome, and low-grade inflammation—exert their effects by acting on stem cells and differentiation progeny toward enteroendocrine cells [78]. In other words, the changes found in the present study are believed to be caused by other factors engaged in the pathophysiology of IBS.

## Conclusion

The present study confirms the finding of a previous study involving another cohort of IBS patients that the density of enteroendocrine cells is reduced in the ileum [36]. This reduction in the enteroendocrine cells seems to be caused by decreases in the clonogenic activity of the stem cell and in the differentiation into enteroendocrine cells from stem-cell progenitors. The changes in the proportions of the endocrine cell types appear to result from switching on and switching off the expressions of certain hormones by the enteroendocrine cells [66–68].

## Abbreviations

CgA: Chromogranin A
GIP: Gastric inhibitory polypeptide
IBS: Irritable bowel syndrome
IBS-C: Constipation-predominant IBS
IBS-D: Diarrhea-predominant IBS
IBS-M: Mixed-diarrhea-and-constipation IBS
Msi-1: Musashi-1
NEUROG3: Neurogenin 3
PP: Pancreatic polypeptide
PYY: Peptide YY
